# Determinants of suboptimal CD4^+^ T cell recovery after antiretroviral therapy initiation in a prospective cohort of acute HIV‐1 infection

**DOI:** 10.1002/jia2.25585

**Published:** 2020-09-19

**Authors:** Ryan Handoko, Donn J Colby, Eugène Kroon, Carlo Sacdalan, Mark de Souza, Suteeraporn Pinyakorn, Peeriya Prueksakaew, Chutharat Munkong, Sasiwimol Ubolyam, Siriwat Akapirat, Jennifer Chiarella, Shelly Krebs, Irini Sereti, Victor Valcour, Robert Paul, Nelson L. Michael, Nittaya Phanuphak, Jintanat Ananworanich, Serena Spudich, the SEARCH 010/RV254 Study Team

**Affiliations:** ^1^ Yale School of Medicine New Haven CT USA; ^2^ SEARCH The Thai Red Cross AIDS Research Centre Bangkok Thailand; ^3^ United States Military HIV Research Program Walter Reed Army Institute of Research Silver Spring MD USA; ^4^ The Henry M. Jackson Foundation for the Advancement of Military Medicine Bethesda MD USA; ^5^ HIV‐NAT The Thai Red Cross AIDS Research Centre Bangkok Thailand; ^6^ Armed Forces Research Institute of Medical Sciences US Army Medical Directorate Bangkok Thailand; ^7^ Laboratory of Immunoregulation National Institute of Allergy and Infectious Diseases National Institutes of Health Bethesda MD USA; ^8^ Memory and Aging Center Department of Neurology University of California San Francisco San Francisco CA USA; ^9^ Missouri Institute of Mental Health University of Missouri‐St. Louis St. Louis MO USA

**Keywords:** ARV, Asia, men who have sex with men, LMIC, immunology, HIV care continuum

## Abstract

**Introduction:**

Up to 30% of individuals treated with antiretroviral therapy (ART) during chronic HIV fail to recover CD4 counts to >500 cells/mm^3^ despite plasma viral suppression. We investigated the frequency and associations of suboptimal CD4 recovery after ART started during acute HIV infection (AHI).

**Methods:**

Participants who started ART in Fiebig I to V AHI with ≥48 weeks of continuous documented HIV‐RNA < 50 copies/mL were stratified by CD4 count at latest study visit to suboptimal immune recovery (SIR; CD4 < 350 cells/mm^3^), intermediate immune recovery (IIR; 350 ≤ CD4 < 500) and complete immune recovery (CIR; CD4 ≥ 500). Clinical and laboratory parameters were assessed at pre‐ART baseline and latest study visit. Additional inflammatory and neurobehavioral endpoints were examined at baseline and 96 weeks.

**Results:**

Of 304 participants (96% male, median 26 years old) evaluated after median 144 (range 60 to 420) weeks of ART initiated at median 19 days (range 1 to 62) post‐exposure, 3.6% (n = 11) had SIR and 14.5% (n = 44) had IIR. Pre‐ART CD4 count in SIR compared to CIR participants was 265 versus 411 cells/mm^3^ (*p* = 0.002). Individuals with SIR or IIR had a slower CD4 rate of recovery compared to those with CIR. Timing of ART initiation by Fiebig stage did not affect CD4 count during treatment. Following ART, the CD8^+^T cell count (*p* = 0.001) and CD4/CD8 ratio (*p* = 0.047) were lower in SIR compared to CIR participants. Compared to the CIR group at week 96, the combined SIR and IIR groups had higher sCD14 (*p* = 0.008) and lower IL‐6 (*p* = 0.04) in plasma, without differences in neuropsychological or psychiatric indices.

**Conclusions:**

Despite immediate and sustained treatment in AHI, suboptimal CD4 recovery occurs uncommonly and is associated with low pre‐ART CD4 count as well as persistent low CD8 count and CD4/CD8 ratio during treatment.

## 1 Introduction

In the era of potent antiretroviral therapy (ART), ongoing HIV replication is adequately suppressed to undetectable levels in appropriately treated individuals. However, up to 30% of ART‐treated individuals fail to achieve CD4^+^ T cell counts to a normal level (>500 cells/mm^3^), despite suppression of HIV replication [1]. This clinical immunologic non‐response to ART has been associated with factors including older age, viral hepatitis coinfection, nadir CD4^+^ T cell count, longer duration of untreated HIV infection and worse morbidity and mortality [1, 2, 3, 4, 5]. Additionally, poor CD4 recovery is more likely to occur in patients who initiate ART late in the course of infection [2, 6].

In early HIV infection, there is a rapid and severe depletion of circulating CD4^+ ^T cells, followed by a spontaneous but temporary recovery [7]. Initiation of ART within four months of HIV infection to coincide with this temporary recovery period has been associated with improved CD4 recovery [2, 8]. Acute HIV infection (AHI) describes the earliest stages of HIV infection, when HIV serology remains non‐reactive or inconclusive yet viral replication is detectable in tissue and blood [9]. Although it is uncommon to identify individuals during this narrow window, the RV254/SEARCH 010 study follows a cohort of individuals with AHI in Thailand who are identified and begin ART soon after HIV infection. Initiation of ART during AHI partially resolves systemic inflammation [10]. Furthermore, initiation of ART during Fiebig stage I (detectable HIV‐RNA while tests for p24 antigen and HIV antibody are negative) results in reduced viral reservoir and improved immunological reconstitution compared to Fiebig stages II‐IV [11].

Whether identifying and treating HIV at the earliest stages of infection (within one month) reduces the frequency of clinical immunologic non‐response is not known, though improved CD4 recovery has been described in ART initiation within four to six months of infection or seroconversion [2, 8, 12]. Additionally, little is known about the dynamics of CD4 recovery with early ART initiation in Asian participant populations [13]. Furthermore, whether HIV‐associated neuroinflammation or HIV‐associated neurocognitive or affective symptoms are associated with poor CD4 recovery has yet to be examined. We investigated if poor CD4 recovery occurs when ART is started during AHI. We identified pre‐ART (baseline) and on‐ART clinical and laboratory parameters associated with poor CD4 recovery, including systemic, central nervous system (CNS) and coinfection factors known to associate with CD4 recovery in chronic infection.

## 2 Methods

### 2.1 Study participants

Adult participants with AHI identified at the Thai Red Cross AIDS Research Centre in Bangkok were enrolled in the ongoing RV254/SEARCH010 study (clinicaltrials.gov NCT00796146), as previously described [14]. Participants were offered immediate initiation of ART via an accompanying protocol (clinicaltrials.gov NCT00796263). Standard first‐line ART through 2016 included efavirenz plus two nucleoside reverse transcriptase inhibitors. Efavirenz could be replaced by ritonavir‐boosted lopinavir or raltegravir for intolerance or resistance. A subset received a five‐drug regimen that added raltegravir and maraviroc [15]. The majority were switched from efavirenz to dolutegravir starting in 2017. Participants underwent serial interviews, examinations and phlebotomy, with optional cerebrospinal fluid (CSF) sampling. Thai HIV‐uninfected controls were male participants from RV304/SEARCH013, a tissue sampling study (NCT01397669) [16]. All participants provided written informed consent prior to enrolment in the cohort. The research protocol was approved by institutional review boards at Chulalongkorn University Hospital, Yale School of Medicine, UCSF and the Armed Forces Research Institute for Medical Sciences.

This analysis included all participants who initiated ART between April 2009 and April 2016 with at least 48 weeks of continuous documented HIV‐RNA < 50 copies/mL, regardless of time to suppression or pre‐ART CD4^+^ T cell count. Eligible participants were stratified by latest CD4^+^ T cell count to suboptimal immune recovery (SIR, <350 cells/mm^3^), intermediate immune recovery (IIR, 350 to 499 cells/mm^3^) and complete immune recovery (CIR, ≥500 cells/mm^3^). None of the participants enrolled in analytic treatment interruption or interventional substudies before the latest follow‐up visit used in the current analysis.

### 2.2 Sampling and laboratory testing

Clinical and laboratory parameters were assessed at baseline and latest study visit. Blood and CSF markers of immune activation, neuropsychological (NP) testing, and mood assessments were examined at a standardized interval of 96 weeks after starting ART. CD4^+^ T cell count and viral load were assessed at all available study visits (weeks 0, 2 and 4; then every four weeks until week 24; then every 12 weeks thereafter) to investigate longitudinal trends.

CD4^+^ T cell count was measured by single‐ and dual‐platform flow cytometry (Becton‐Dickinson). HIV‐RNA in plasma and CSF was performed using the COBAS AMPLICOR HIV‐1 Monitor Test v1.5 or COBAS Taqman HIV‐1 Test v2.0 (Roche Molecular Systems). Plasma soluble CD14 (sCD14), intestinal fatty acid binding protein (I‐FABP) (R&D Systems) and hyaluronic acid (Corgenix) were measured by ELISA. C‐reactive protein was measured by electrochemiluminescence assay (Meso Scale Discovery). D‐dimer was measured by enzyme‐linked fluorescent assay (bioMerieux). Tumour necrosis factor‐alpha (TNF‐alpha) and high‐sensitivity interleukin 6 (IL‐6) were measured by the Luminex platform (Millipore). All assays for biomarkers were performed in duplicate on cryopreserved acid citrate dextrose plasma for research purposes only following a single thaw with the exception of I‐FABP (two thaws). Anti‐hepatitis C antibodies, hepatitis B surface antigen (HBsAg), and anti‐HBsAg antibodies were measured by chemiluminescent microparticle immunoassay (Abbott). Syphilis testing was measured by B VDRL Antigen BD Difco (Becton Dickinson), Macro‐Vue RPR Card Tests (Becton Dickinson) and Serodia TPPA (Fujirebio Diagnostics).

### 2.3 Statistical analysis

Data were reported as median (interquartile range, IQR) values, except when otherwise indicated. Comparisons between CD4 recovery groups were performed using the Mann–Whitney *U* test (comparing SIR to CIR, or combined SIR and IIR to CIR) or Kruskal–Wallis test (when comparing across all three groups) for continuous variables, χ^2^ test for categorical variables, and linear regression for slope analyses. Slope analyses were performed by assessing CD4 change from baseline to week 48 on ART, divided by 48; a univariate linear regression model was used to assess whether the CD4 recovery rate differed between the CD4 recovery groups. Univariate and multivariate logistic regression analyses were performed to identify factors associated with CD4 recovery < 500 cells/mm^3^. Factors with a *p* ≤ 0.10 in univariate analyses were included in the backward stepwise multivariate analysis to select the final model with probability to enter = 0.01 and probability to retain = 0.05. The final model was also adjusted for ART duration. Statistical tests were two‐sided, and differences were considered significant at *p* < 0.05. Differences were considered suggestive of a trend at *p* < 0.10. Analyses were performed using Stata (version 15; StataCorp), R (version 3.6.1; R Foundation for Statistical Computing), and Prism (version 7.0; GraphPad) software.

## 3 Results

### 3.1 Study participant characteristics

During the RV254/SEARCH010 study period, 304 participants with AHI immediately started ART and had documented viral load < 50 copies/mL for at least 48 weeks (Figure S1), of whom 79 underwent optional CSF sampling. Five participants died and 15 were lost to follow‐up. 96% of enrolees were Thai men, the majority men who have sex with men. Median age was 26 years (range 18 to 57). ART was started at a median 19 days post‐estimated infection (range 1 to 62). Median latest follow‐up visit was at 144 weeks (IQR 108 to 216, range 60 to 420), which also represents the median duration of ART.

### 3.2 **CD**4^+^ **T cell recovery after ART in acute HIV**

Of the 304 participants, the most recent CD4 count was < 350 cells/mm^3^ (SIR) in 3.6%, 350 to 499 cells/mm^3^ (IIR) in 14.5% and ≥ 500 cells/mm^3^ (CIR) in 81.9%. Most recent CD4 count was median 667 cells/mm^3^ (IQR 551 to 810). Twenty‐three male HIV‐negative Thai controls had a median CD4 count of 924 cells/mm^3^ (IQR 687 to 1103) (Table S1). Viral load at enrolment, time from HIV transmission to ART initiation, week of first documented viral suppression, and Fiebig stage at enrolment did not differ between recovery groups (Table 1). Duration of ART was shortest in the IIR group (median 120 weeks, IQR 84 to 192, *p* = 0.03) but did not differ between SIR (156, 84 to 180) and CIR groups (156, 108 to 216, *p* = 0.5). Duration of ART was shorter in the combined suboptimal and intermediate recovery group versus CIR (*p* = 0.01).

**Table 1:** Characteristics of participants treated in acute HIV infection stratified by immunologic recovery

Characteristics	Suboptimal immune recovery, CD4 < 350, (n = 11)	Intermediate immune recovery, 350 ≤ CD4 < 500 (n = 44)	Complete immune recovery, CD4 ≥ 500 (n = 249)	*p*‐value
Age, years (IQR)	23 (20 to 30)	26 (23 to 30)	26 (23 to 33)	0.3
Sex, male:female, n	11:0	44:0	237:12	0.3
Risk behaviour, n (%)				
WSM	0 (0)	0 (0)	12 (5)	0.4
MSW	1 (9)	3 (7)	9 (4)	
MSM	10 (91)	41 (93)	228 (92)	
Thai ethnicity, n (%)	11 (100)	43 (98)	244 (98)	0.9
Illicit drug use during HIV exposure, n (%)	2 (18)	13 (30)	51 (21)	0.4
HIV‐RNA at enrolment (log_10_ copies/mL), median (IQR)	5.9 (5.4 to 7.3)	6.1 (5.4 to 6.9)	5.7 (5.2 to 6.6)	0.3
Time to ART initiation (days), median (IQR)	20 (14 to 23)	21 (15 to 28)	19 (15 to 26)	0.6
1^st^ week of viral suppression, median (IQR)	16 (8 to 24)	12 (8 to 24)	12 (8 to 24)	>0.9
ART duration (weeks), median (IQR)	156 (84 to 180)	120 (84 to 192)	156 (108 to 216)	0.03
Fiebig stage at enrolment, n (%)				
Stage I	2 (18)	5 (11)	44 (18)	0.2
Stage II	4 (36)	9 (21)	71 (29)	
Stage III	5 (45)	22 (50)	90 (36)	
Stage IV	0 (0)	6 (14)	26 (10)	
Stage V	0 (0)	2 (5)	18 (7)	

HIV, human immunodeficiency virus; WSM, women who have sex with men; MSW, men who have sex with women; MSM, men who have sex with men; IQR, interquartile range.

**Figure 1:**
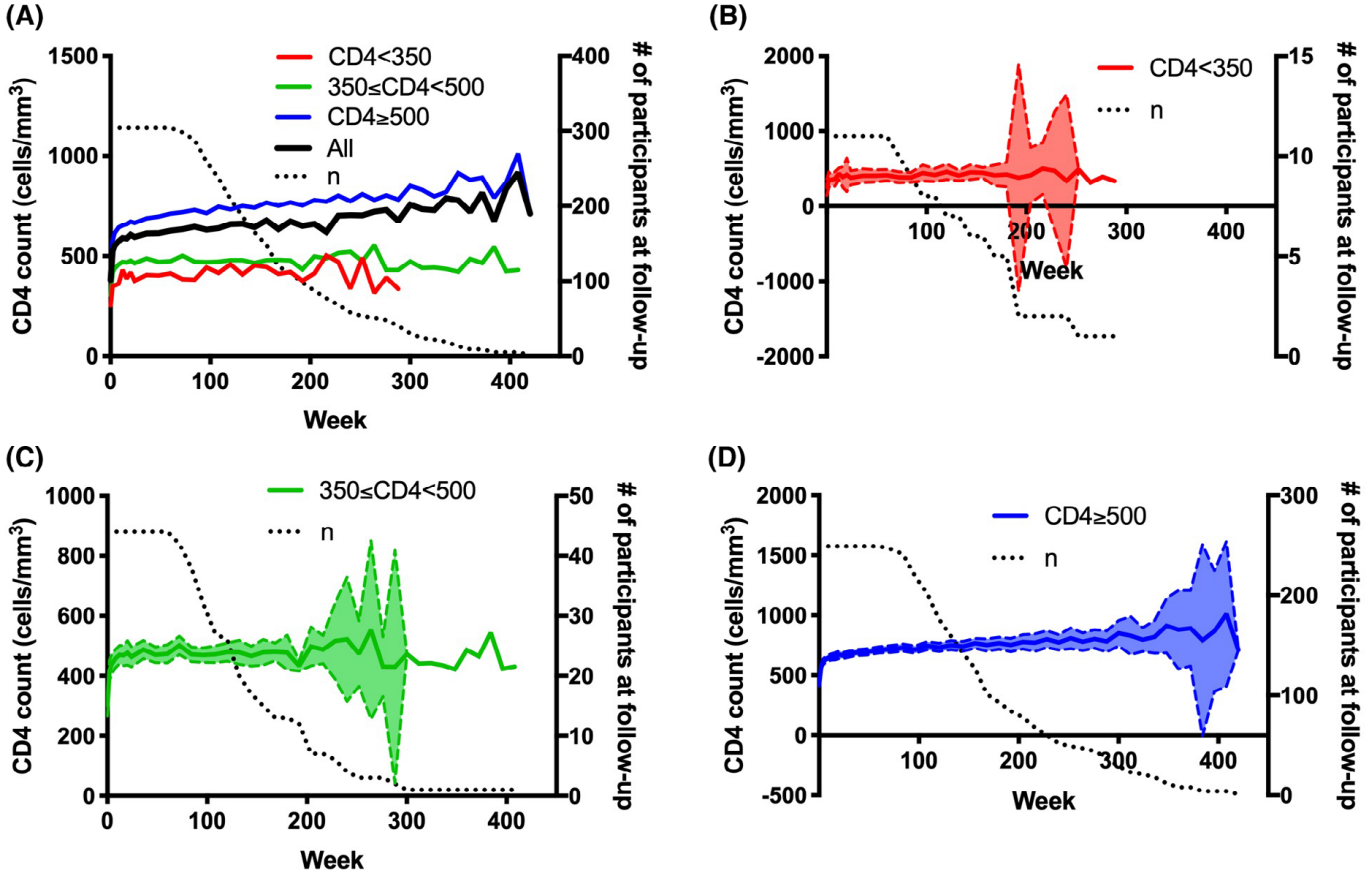
Longitudinal CD4^+^ T cell counts of participants with acute HIV infection according to suboptimal, intermediate and complete immune recovery groups. **(A)** Mean CD4^+^ T cell counts of all participants (black) and by suboptimal (red), intermediate (green) and complete immune recovery (blue) groups. Dotted black line represents the total number of participants across all recovery groups at each study visit week. **B**‐**D**, CD4^+^ T cell counts of participants in suboptimal **(B)**, intermediate **(C)** and complete **(D)** recovery groups. Solid lines represent the mean CD4^+^ T cell count at each follow‐up study visit week. Dashed lines represent the 95% confidence interval of CD4^+^ T cell counts at each study visit week. Dotted black lines represent the total number of participants in each respective recovery group at each study visit week

CD4 counts of individuals in the three recovery groups were plotted at each study visit week (Figure 1), using median 18 observations (IQR 15 to 24). Using slope analysis, mean (standard deviation) rates of recovery were 3.1 (3.4), 3.5 (2.1) and 5.5 (4.1) cells/mm^3^/week for SIR, IIR and CIR groups respectively. Recovery rate in IIR was significantly slower compared to complete recovery (rate difference − 2.0 cells/mm^3^/week, 95% confidence interval [CI] −3.3 to − 0.8, *p* = 0.002). There was a trend of slower rate in SIR compared to CIR (rate difference − 2.4 cells/mm^3^/week, 95% CI − 4.7 to 0.0, *p* = 0.05). There was no difference between SIR and IIR groups. Individuals were stratified into groups of low (<350 cells/mm^3^), medium (350 to 499 cells/mm^3^) and high (≥500 cells/mm^3^) baseline CD4 count, and longitudinal CD4 counts within each group were plotted at each study visit week (Figure S2). Individuals were additionally stratified into groups of low (<350 cells/mm^3^), medium (350 to 499 cells/mm^3^) and high (≥500 cells/mm^3^) concurrent CD4 count, and proportions of each group at intervals of 24 weeks were displayed (Figure S3).

**Figure 2:**
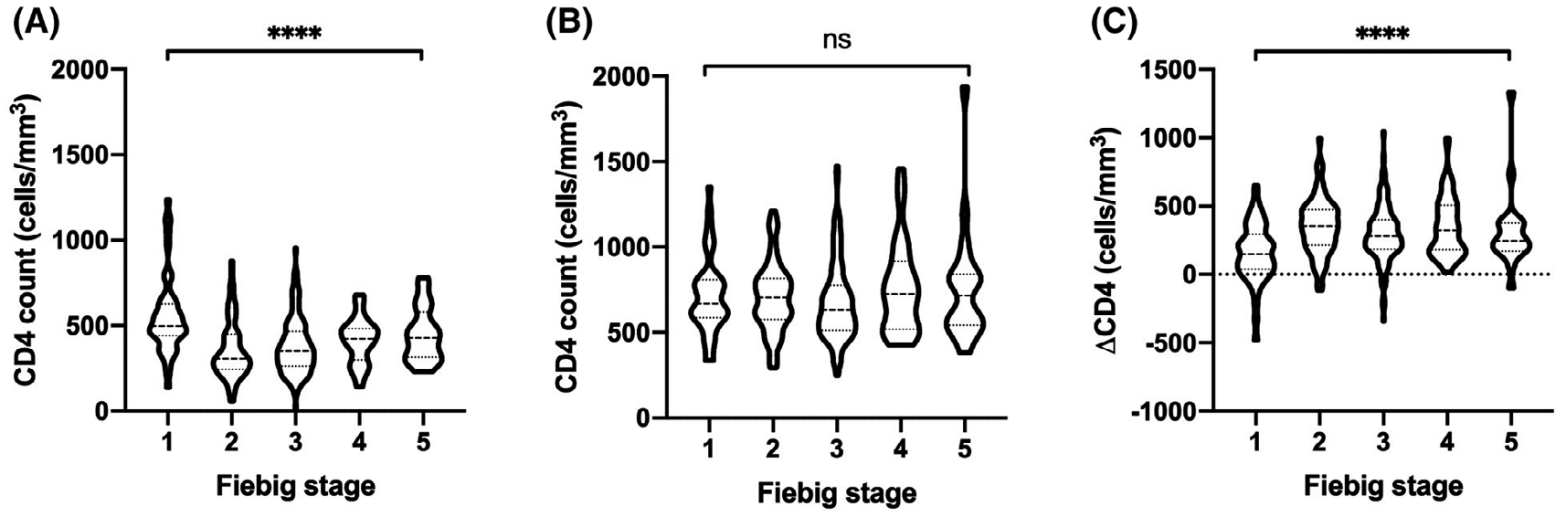
CD4^+^ T cell count by Fiebig stage at enrolment. Violin plots of CD4 count at baseline **(A)** and at latest study visit **(B)** by Fiebig stage at enrolment. **(C)**, Violin plot of change in CD4 count between baseline and latest study visit by Fiebig stage at enrolment. Comparison between groups by Kruskal–Wallis test. ****p < 0.0001, ns indicates not statistically significant

CD4 count differed by Fiebig stage at enrolment and was highest for Fiebig stage I at enrolment (*p* < 0.0001, Figure 2). CD4 count at latest study visit was not different by Fiebig stage at enrolment. Change in CD4 count between baseline and latest study visit was different by Fiebig stage at enrolment and was greatest for Fiebig stage II and least for Fiebig stage I at enrolment (*p* < 0.0001).

### 3.3 Baseline pre‐ART predictors of CD4 recovery after ART

At baseline, CD4 and absolute lymphocyte counts were lower in participants with SIR compared to CIR (CD4 count: median 265 vs. 411 cells/mm^3^, *p* = 0.002, Table 2). CD8 count, CD4/CD8 ratio, haemoglobin and platelet count at baseline were not different between SIR and CIR.

**Table 2:** Pre‐ART baseline predictors of immunological recovery

Pre‐ART baseline predictors	Suboptimal immune recovery, CD4 < 350 (n = 11)	Suboptimal & Intermediate Immune Recovery, CD4 < 500 (n = 55)	Complete immune recovery, CD4 ≥ 500 (n = 249)	*p*‐value^a^	*p*‐value^b^
CD4 count at baseline (cells/mm^3^)	265 (91 to 371)	275 (214 to 371)	411 (303 to 533)	0.002	<0.001
CD8 count at baseline (cells/mm^3^)	412 (228 to 575)	448 (298 to 847)	512 (339 to 854)	0.09	0.4
CD4/CD8 ratio at baseline	0.67 (0.37 to 0.94)	0.66 (0.35 to 0.87)	0.81 (0.47 to 1.2)	0.4	0.003
WBC count at baseline (×10^3^ cells/mm^3^)	4.5 (3 to 5.6)	4.7 (4.0 to 5.6)	5.5 (4.4 to 7.0)	0.03	0.003
Absolute neutrophil count at baseline (×10^3^ cells/mm^3^)	2.84 (1.56 to 3.52)	2.90 (2.24 to 3.60)	3.13 (2.18 to 4.22)	0.3	0.2
Absolute lymphocyte count at baseline (×10^3^ cells/mm^3^)	1.06 (0.69 to 1.48)	1.18 (0.93 to 1.91)	1.58 (1.21 to 2.10)	0.003	<0.001
Monocyte count at baseline (×10^3^ cells/mm^3^)	0.38 (0.28 to 0.63)	0.40 (0.31 to 0.52)	0.52 (0.42 to 0.66)	0.1	<0.001
Eosinophil count at baseline (×10^3^ cells/mm^3^)	0.045 (0.000 to 0.308)	0.023 (0.012 to 0.049)	0.038 (0.018 to 0.082)	0.9	0.09
Haemoglobin at baseline (g/dL)	15 (14 to 16)	15 (14 to 16)	15 (14 to 16)	0.2	0.2
Platelet count at baseline (×10^3^/mm^3^)	215 (167 to 233)	215 (180 to 248)	234 (188 to 296)	0.1	0.01
sCD14 (µg/L)	–	1.3 (1.1 to 2.3)^c^	1.6 (1.1 to 1.9)^d^	–	0.9
IL‐6 (pg/mL)	–	0.6 (0.4 to 1.4)^e^	0.5 (0.3 to 0.9)^f^	–	0.3
Hyaluronic acid (ng/mL)	–	17.6 (9.7 to 30.2)^c^	18.0 (9.0 to 32.4)^d^	–	0.96
Intestinal fatty acid binding protein (pg/mL)	–	738 (343 to 944)^c^	1044 (637 to 1559)^d^	–	0.045
C‐reactive protein (mg/L)	–	2.1 (0.5 to 4.2)^c^	1.3 (0.7 to 3.4)^d^	–	0.96
TNF‐alpha (pg/mL)	–	1.3 (0.8 to 2.1)^e^	1.2 (0.7 to 2.2)^f^	–	0.9
D‐dimer (ng/mL)	–	357 (283 to 473)^c^	266 (173 to 435)^d^	–	0.3

Median (IQR). CD4, CD4^+^ T cell; CD8, CD8^+^ T cell; WBC, white blood cell; sCD14, soluble CD14; IL‐6, interleukin‐6; TNF‐alpha, tumour necrosis factor alpha.

^a^ P‐value is for suboptimal immune recovery versus complete immune recovery

^b^ P‐value is for combined suboptimal & intermediate immune recovery versus complete immune recovery

^c^ n = 8

^d^ n = 63

^e^ n = 16

^f^ n = 118.

Given the small size of the SIR group and the similar CD4 trajectories of the SIR and IIR groups, we combined these two groups and compared baseline predictors with complete recovery. CD4/CD8 ratio and CD4^+^ T cell, total WBC, absolute lymphocyte, absolute monocyte and platelet counts were lower in the combined group compared to the CIR group at baseline. I‐FABP was lower in the combined group compared to CIR. Other markers of inflammation were similar between these groups (Table 2).

CSF sample results for SIR (CSF n = 3) and IIR (CSF n = 7) groups were also combined for comparison to CIR (CSF n = 69). CSF viral load, WBC count and glucose were not different between the combined and CIR groups. Baseline CSF neopterin trended in elevation in the combined group (n = 10) compared to the CIR group (n = 69, median 2938 vs. 1623 pg/mL, *p* = 0.05). SIR versus CIR groups did not differ on neuropsychological tests or psychiatric indices at baseline (Table S2). Markers for co‐infection or exposure to hepatitis B virus, hepatitis C virus and syphilis at baseline were not associated with CD4 recovery (Table S2).

### 3.4 On‐ART factors associated with CD4 recovery

The ART regimen initiated at AHI did not predict CD4 recovery (Table S4). By the time of most recent study visit, 113 of the 304 participants (37%) were switched to a dolutegravir‐containing regimen after a median (IQR) duration of ART of 145 (107 to 212) weeks. Participants switched to a dolutegravir‐containing regimen were on dolutegravir for a median 10 weeks (IQR 3 to 12). There was no association between switch to dolutegravir and CD4 recovery (not shown).

On ART, CD8^+^ T cell count and CD4/CD8 ratio were lower in SIR compared to CIR (median 318 vs. 621 cells/mm^3^, IQR 279 to 628 vs. 490 to 843, *p* = 0.001 and median 1.05 vs. 1.18, IQR 0.47 to 1.20 vs. 0.91 to 1.48, *p* = 0.047 respectively) (Table 3). Additionally, haemoglobin was higher in SIR compared to CIR. Platelets were diminished in the SIR group.

**Table 3:** On‐ART variables associated with immunological recovery

On‐ART variables	Suboptimal immune recovery, CD4 < 350 (n = 11)	Suboptimal & intermediate immune recovery, CD4 < 500 (n = 55)	Complete immune recovery, CD4 ≥ 500 (n = 249)	*p*‐value^a^	*p*‐value^b^
CD8 count at latest visit	318 (279 to 628)	460 (359 to 600)	621 (490 to 843)	0.001	<0.001
CD4/CD8 ratio	1.05 (0.47 to 1.20)	0.95 (0.76 to 1.16)	1.18 (0.91 to 1.48)	0.047	<0.001
WBC count (× 10^3^ cells/mm^3^)	4.88 (4.68 to 6.25)	4.99 (4.36 to 5.74)	6.13 (5.34 to 7.27)	0.02	<0.001
Absolute neutrophil count at latest week (×10^3^ cells/mm^3^)	3.37 (2.52 to 4.45)	2.92 (2.27 to 3.57)	3.23 (2.48 to 4.21)	0.9	0.07
Absolute lymphocyte count at latest week (×10^3^ cells/mm^3^)	0.99 (0.91 to 1.40)	1.46 (1.24 to 1.67)	2.19 (1.85 to 2.48)	2 × 10^−7^	<0.001
Monocyte count at latest week (×10^3^ cells/mm^3^)	0.41 (0.36 to 0.64)	0.41 (0.35 to 0.51)	0.51 (0.40 to 0.62)	0.3	0.001
Eosinophil count at latest week (×10^3^ cells/mm^3^)	0.13 (0.09 to 0.19)	0.13 (0.09 to 0.20)	0.13 (0.08 to 0.24)	1.0	0.7
Haemoglobin (g/dL)	15.5 (14.5 to 16.4)	15.1 (14.4 to 15.8)	14.7 (14.0 to 15.6)	0.02	0.04
Platelet count (×10^3^/mm^3^)	223 (185 to 283)	252 (218 to 277)	273 (237 to 313)	0.03	0.002
sCD14 at week 96 (µg/L)^c^	–	1.7 (1.6 to 1.8)^j^	1.1 (1.0 to 1.2)^e^	–	0.008
IL‐6 at week 96 (pg/mL)^c^	–	0.13 (0.11 to 0.56)^f^	0.56 (0.14 to 0.97)^g^	–	0.04
Hyaluronic acid at week 96 (ng/mL)^c^	–	15.1 (13.6 to 21.9)^d^	11.8 (9.0 to 18.9)^e^	–	0.4
Intestinal fatty acid binding protein at week 96 (pg/mL)^c^	–	1533 (1212 to 2058)^d^	2953 (1366 to 3823)^e^	–	0.3
C‐reactive protein (mg/L)	–	0.22 (0.19 to 0.54)^d^	0.49 (0.19 to 1.08)^e^	–	0.7
TNF‐alpha (pg/mL)	–	0.93 (0.40 to 16.13)^f^	1.61 (0.25 to 4.28)^g^	–	0.6
D‐dimer (ng/mL)	–	190 (179 to 401)^d^	138 (102 to 256)^e^	–	0.3

Median (IQR). Factors were measured at latest available study visit unless otherwise indicated. Median study visit weeks were 156 for SIR, 120 for IIR and 156 for CIR. CD4, CD4^+^ T cell; CD8, CD8^+^ T cell; WBC, white blood cell; sCD14, soluble CD14; IL‐6, interleukin‐6; TNF‐alpha, tumour necrosis factor alpha.

^a^ P‐value is for suboptimal immune recovery versus complete immune recovery

^b^ P‐value is for combined suboptimal & intermediate immune recovery versus complete immune recovery

^c^ Factors were measured at a standardized interval of 96 weeks

^d^ n = 3

^e^ n = 25

^f^ n = 8

^g^ n = 66.

After combining suboptimal and intermediate immune recovery groups, we identified on‐ART factors that differed between the combined and CIR groups (Table 3). Consistent with the differences between SIR and CIR, CD8 count, CD4/CD8 ratio and WBC count were lower in the combined group compared to CIR. On‐ART haemoglobin was higher in the combined group, and platelet count was lower. Absolute lymphocyte and monocyte counts were lower in the combined group. Serum sCD14 at week 96 was elevated and IL‐6 was lower in the combined group.

Optional CSF sample size during ART was too small to assess associations between neuroinflammatory markers and CD4 recovery. Suboptimal and complete recovery did not differ in neuropsychological test performance or psychiatric indices at 96 weeks of ART (Table S3). On ART, markers for co‐infection or exposure to hepatitis B virus, hepatitis C virus and syphilis were not associated with CD4 recovery (Table S3). CD4 recovery groups were too small to perform logistic regression analysis using CD4 count <350 cells/mm^3^ as the primary endpoint. In a supplemental analysis, we used multivariate logistic regression analysis to adjust for duration of ART, baseline HIV‐RNA and baseline CD4 count, assessing for CD4 count <500 cells/mm^3^ as a secondary endpoint (Table S5). After adjustment, odds of poor CD4 recovery <500 cells/mm^3^ were higher in participants with baseline CD4/CD8 ratio <1 (odds ratio [OR] 5.62, 95% confidence interval [CI] 1.99 to 15.89, *p* = 0.001), on‐ART CD8 count <500 cells/mm^3^ (OR 4.15, 95% CI 1.94 to 8.88, *p* < 0.001) and on‐ART platelet count <300,000 per mm^3^ (OR 2.94, 95% CI 1.05 to 8.22, *p* = 0.04).

## 4 Discussion

We found that suboptimal CD4 recovery occurs in a small subset of individuals despite treatment in the earliest stages of AHI. Whereas 70% to 85% of individuals with chronic HIV infection had CD4 recovery to >350 cells/mm^3^ after ART, we observed a larger proportion (96.4%) in AHI [1]. A larger proportion of individuals were categorized as intermediate immune responders (CD4 count 350 to 499 cells/mm^3^) as compared to suboptimal immune responders (<350 cells/mm^3^). Prior work has identified determinants of CD4 recovery in early seroconversion, though in the setting of delayed ART initiation [8, 17]. To our knowledge, our study is the first to examine determinants of CD4 recovery with immediate ART initiation in the earliest stages of HIV infection. Furthermore, few studies have identified determinants of CD4 recovery in chronic HIV infection in an Asian population, and none in early seroconversion or primary HIV infection to our knowledge [18, 19, 20].

The degree of lymphatic tissue fibrosis has been shown to correlate with CD4 recovery [21, 22]. As an indicator of a systemic profibrotic state, hyaluronic acid was lower in treated AHI compared to treated chronic HIV infection, yet both remain elevated compared to HIV‐uninfected controls [10]. Taken together, immediate ART initiation in AHI may limit the extent of lymphatic tissue fibrosis compared to that seen in chronic HIV infection, which may explain the lower proportion of suboptimal CD4 recovery in AHI.

When examining the SIR and IIR groups throughout the course of treatment (Figure 1), CD4 count trajectories are similar. Additionally, individuals with IIR appear to have transient decreases in CD4 count below 350 cells/mm^3^, and may in fact sustain an overall CD4 response around or above the threshold of 350 cells/mm^3^. Thus, differences between the combined suboptimal and intermediate recovery group versus CIR are more stark and reveal a state of perturbed immunological recovery as compared to differences between SIR versus IIR.

Poor CD4 recovery (<500 cells/mm^3^) is associated with nadir CD4 count, and thus increases from substantially low nadir may not be captured using an absolute threshold as a definition of CD4 recovery. However, our slope analyses show that individuals with CIR have a higher recovery rate over a fixed time interval of 48 weeks, indicating greater absolute and relative increases in CD4 count. Other definitions of CD4 recovery have accounted for low nadir, such as an increase in CD4 count ≥30% from baseline [23]. We used prior definitions of CD4 recovery as absolute thresholds ≥350 or ≥500 cells/mm^3^ to allow for direct comparison to results in chronic and primary HIV infection. Furthermore, absolute CD4^+^ T cell count during treated HIV infection is a critical clinical measure for risk of opportunistic infections.

Consistent with a prior description of our cohort, individuals diagnosed and started on ART at Fiebig stage I have a higher baseline CD4 count compared to stages II‐IV, with normalization of CD4 count after at least 48 weeks of treatment [11]. Individuals starting on ART at Fiebig stage I have a lower absolute CD4 increase compared to other stages, suggesting that CD4 decline to nadir occurs at later Fiebig stages but is reversible with immediate ART initiation. Strategically timing earlier ART initiation during an interval of CD4 recovery has been associated with better long‐term CD4 recovery [2, 3]. Conversely, ART initiation during an interval of CD4 decline is associated with poor CD4 recovery [24], suggesting that strategic timing of early ART must consider not only nadir CD4 count but also the dynamics of pre‐ART CD4 recovery. However, initiation of ART in Fiebig stages II‐IV did not affect on‐ART CD4 count, suggesting that ART initiation in AHI does not need to be strategically timed and should be prompt.

Interestingly, our study showed that lower CD8 count during treatment, but not at baseline, was associated with poor CD4 recovery, consistent with a previous report [25]. This may indicate suppression of thymic output that has been observed to occur in the setting of poor CD4 recovery [26]. However, another study found that higher baseline CD4 count and lower CD8 count were independently associated with improved CD4 recovery [27]. The CD8 count may thus be interpreted as an indicator of thymic output as well as of immune activation.

Poor CD4 recovery was independently associated with lower CD4/CD8 ratio both at baseline and during treatment in AHI. Reduced CD4/CD8 ratio has been found to be a marker for immune dysregulation and activation in the setting of HIV infection that has been associated with an increased risk for non‐AIDS related morbidity and mortality, including age‐related inflammation and cardiovascular disease [28, 29, 30]. For our group of poor responders treated during AHI, CD4/CD8 ratio at baseline was higher than in participants treated during chronic HIV infection, reflecting less immune dysregulation at the earliest stages of infection [23, 27, 30]. Initiating ART during primary versus chronic HIV infection leads to a higher frequency of CD4/CD8 ratio >1, with higher CD4/CD8 ratio at baseline associated with a lower risk of poor CD4 recovery [3]. An overall faster time to treatment initiation results in improved CD4/CD8 ratio [31]. Interestingly, despite the rapid initiation of ART in early AHI, other markers of chronic inflammation persist in the RV254/SEARCH010 cohort [10].

We analysed markers for microbial translocation and systemic inflammation, finding elevated sCD14 in suboptimal CD4 responders after treatment. Some prior studies show increased sCD14, its ligand lipopolysaccharide (LPS) [32], bacterial 16S ribosomal DNA [33] or neutrophil infiltration [34] in poor CD4 recovery, whereas others find no differences [23]. Surprisingly, in our study IL‐6 was elevated in complete recovery. Elevated IL‐6 has been associated with increased risk of HIV‐associated opportunistic disease [35].

Platelet counts were lower in poor compared to complete recovery both at baseline and after ART, consistent with a prior study [36]. HIV‐associated thrombocytopenia has several possible mechanisms, including immune‐mediated destruction, decreased survival, decreased production and infection of megakaryocytes [37].

Poor CD4 recovery trended towards association with neuroinflammation at baseline, as measured by CSF neopterin. To our knowledge, this is the first study assessing the association between neuroinflammation and impaired CD4 recovery, though other CNS biomarkers have been associated with CD4 recovery [38]. Elevated CSF neopterin has been associated with CSF HIV‐RNA and neurological complications of HIV infection [39]. Elevated plasma neopterin has been associated with poor CD4 recovery in treated HIV infection [40].

Our study has several limitations. The number of participants with suboptimal CD4 recovery was low and limits the identification and assessment of associated factors. On‐ART factors were measured at the week of the latest available study visit, which was lower in the combined suboptimal and intermediate recovery group compared to CIR. Thus, the overall longer treatment duration in the complete recovery group possibly confounds the finding of improved CD4^+^ T cell gains. However, our slope analysis examines rate of recovery over a fixed time interval, showing that the CIR group exhibits both higher and faster CD4 recovery. Our study did not have sufficient numbers of lumbar punctures after 48 weeks of ART and thus cannot assess the degree of neuroinflammation after ART in poor responders. The participants in this study are young and may have a lower baseline inflammatory state due to young age. Though some prior investigations have suggested the HIV‐uninfected Thai population have a lower CD4^+^ T cell count reference range compared to predominantly white or European reference populations [41], even after long‐term ART, our participants with AHI did not recover CD4 counts to that of HIV‐negative controls. Finally, we investigated a large number of possible factors that may influence CD4 recovery in treated AHI. Though there is the possibility of spurious findings, our findings are largely in agreement with prior reports from primary and chronic HIV infection.

## 5 Conclusions

In conclusion, suboptimal CD4 recovery following initiation of ART during AHI is low (<5%) compared to chronic HIV infection and is characterized by a low CD4 count at baseline and persistent low CD8 count during treatment. Poor recovery is also associated with lower CD4/CD8 ratio during treatment. Future work should be directed at determining whether ART should be initiated as early as possible in AHI versus strategic timing of ART within AHI to coincide with spontaneous CD4 recovery in order to optimize long‐term CD4 recovery [2].

## Competing Interests

VV has consulted for Merck and ViiV Healthcare. JA had previously received honoraria from Merck, ViiV Healthcare, Roche, AbbVie and Gilead for her participation in advisory meetings. SS directs a study within the AIDS Clinical Trials Group that receives study medications from ViiV Healthcare. The remaining authors report no relevant conflicts of interest.

## Authors’ Contributions

RH, SS and JA conceived the study, participated in its design, coordination and statistical analysis, and participated in drafting of the manuscript. DC and EK participated in study design and coordination. CS, MDS, PP and CM participated in study coordination. SP and JC participated in data management and statistical analysis. SU and SA supervised the research laboratories. SK supervised the biomarker assays. IS participated in drafting of the manuscript. VV and RP supervised neuropsychological testing. NM, NP and JA oversaw the study cohort. All authors read and approved the final manuscript.

## Author Information

Results from this study were presented previously in part in: Handoko R, Colby D, Kroon E, de Souza M, Pinyakorn S, Prueksakaew P, Chiarella J, Krebs S, Sereti I, Valcour V, Michael N, Phanuphak N, Ananworanich J, Spudich S. Determinants of suboptimal immunological response after ART initiation in acute HIV. Conference on Retroviruses and Opportunistic Infections, 4 to 7 March 2018, Boston, MA.

## Abbreviations

## Supporting information


**Table S1.** Characteristics of Thai participants treated in acute HIV infection and of HIV‐negative control participants
**Table S2.** Additional pre‐ART baseline predictors of CD4+ T cell recovery, including co‐infection biomarkers, neuropsychological testing, and depression screening
**Table S3.** Additional on‐ART variables associated with CD4+ T cell recovery, including co‐infection biomarkers, neuropsychological testing, and depression screening
**Table S4.** Antiretroviral therapy regimen at initiation in acute HIV infection, stratified by CD4+ T cell recovery
**Table S5.** Multivariate logistic regression analysis assessing likelihood of CD4 count < 500 cells/mm3, adjusting for duration of ART, baseline HIV‐RNA, and baseline CD4 count
**Figure S1.** Longitudinal HIV‐RNA of participants with acute HIV infection.
**Figure S2.** Longitudinal CD4^+^ T cell counts of participants with acute HIV infection according to low, medium, and high baseline CD4 count.
**Figure S3.** Proportions of low, medium, and high concurrent CD4^+^ T cell counts of participants with acute HIV infection.
