# Supplementary material for: Determinants of suboptimal CD4+ T cell recovery after antiretroviral therapy initiation in a prospective cohort of acute HIV‐1 infection
Source: J Int AIDS Soc. 2020 Sep 19;23(9):e25585. doi: 10.1002/jia2.25585 (PMC7507109; doi:10.1002/jia2.25585)
Supplement: Supplementary file 1 — Table S1. Characteristics of Thai participants treated in acute HIV infection and of HIV‐negative control participants Table S2. Additional pre‐ART baseline predictors of CD4+ T cell recovery, including co‐infection biomarkers, neuropsychological testing, and depression screening Table S3. Additional on‐ART variables associated with CD4+ T cell recovery, including co‐infection biomarkers, neuropsychological testing, and depression screening Table S4. Antiretroviral therapy regimen at initiation in acute HIV infection, stratified by CD4+ T cell recovery Table S5. Multivariate logistic regression analysis assessing likelihood of CD4 count < 500 cells/mm3, adjusting for duration of ART, baseline HIV‐RNA, and baseline CD4 count Figure S1. Longitudinal HIV‐RNA of participants with acute HIV infection. Figure S2. Longitudinal CD4+ T cell counts of participants with acute HIV infection according to low, medium, and high baseline CD4 count. Figure S3. Proportions of low, medium, and high concurrent CD4+ T cell counts of participants with acute HIV infection. [file JIA2-23-e25585-s001.docx]

Supporting Information for:

**Determinants of Suboptimal CD4+ T Cell Recovery After Antiretroviral Therapy Initiation in a Prospective Cohort of Acute HIV-1 Infection**

Ryan Handoko^1^, Donn J. Colby^2^, Eugène Kroon^2^, Carlo Sacdalan^2^, Mark de Souza^2^, Suteeraporn Pinyakorn^2-4^, Peeriya Prueksakaew^2^, Chutharat Munkong^2^, Sasiwimol Ubolyam^5^, Siriwat Akapirat^6^, Jennifer Chiarella^1^, Shelly Krebs^3,4^, Irini Sereti^7^, Victor Valcour^8^, Robert Paul^9^, Nelson L. Michael^3^, Nittaya Phanuphak^2^, Jintanat Ananworanich^2-4^, Serena Spudich^1§^, on behalf of the SEARCH 010/RV254 Study Team

1. Yale School of Medicine, New Haven, CT, USA
2. SEARCH, The Thai Red Cross AIDS Research Centre, Bangkok, Thailand
3. United States Military HIV Research Program, Walter Reed Army Institute of Research, Silver Spring, MD, USA
4. The Henry M. Jackson Foundation for the Advancement of Military Medicine, Bethesda, MD, USA
5. HIV-NAT, The Thai Red Cross AIDS Research Centre, Bangkok, Thailand
6. Armed Forces Research Institute of Medical Sciences, US Army Medical Directorate, Bangkok, Thailand
7. Laboratory of Immunoregulation, National Institute of Allergy and Infectious Diseases, National Institutes of Health, Bethesda, MD, USA
8. Department of Neurology, University of California San Francisco Memory and Aging Center, Sandler Neurosciences Center, San Francisco, CA, USA
9. Missouri Institute of Mental Health, University of Missouri-St. Louis, St. Louis, MO, USA

^§^ Corresponding author:

Serena Spudich, MD

Professor, Department of Neurology

Yale University School of Medicine

300 George Street, Room 8300c

New Haven, Connecticut, 06511 USA

Tel: 1-650-400-1222

Email: serena.spudich@yale.edu

**Supplemental Table 1.**

| Characteristics | HIV Uninfected (n=23) | Acute HIV Infection (n=304) |
| --- | --- | --- |
| Age (years), median (IQR) | 30 (26-33) | 26 (23-32) |
| Gender, *n* (%)  Male  Female | 23 (100)  0 (0) | 292 (96)  12 (4) |
| CD4+ T cell count at week 0 (cells/mm^3^), median (IQR) | 924 (687-1103) | 381 (275-504) |
| CD4+ T cell count at latest visit (cells/mm^3^), median (IQR) | - | 667 (551-810) |

**Supplemental Table 2.**

| Pre-ART Baseline Predictors | Suboptimal Recovery, CD4<350 | Suboptimal & Intermediate Recovery, CD4<500 | Complete Recovery, CD4≥500 | p-value^†^ | p-value^‡^ |
| --- | --- | --- | --- | --- | --- |
| Positive anti-HCV, *n* (%) | 0/10 (0) | 1/54 (2) | 2/248 (1) | 0.8 | 0.5 |
| Positive anti-HBs, *n* (%) | 5/11 (45) | 28/54 (52) | 133/249 (53) | 0.6 | 0.8 |
| Positive HBsAg, *n* (%) | 1/11 (9) | 3/55 (5) | 15/249 (6) | 0.7 | 0.9 |
| Reactive VDRL, *n* (%) | 2/10 (20) | 7/54 (13) | 30/249 (12) | 0.5 | 0.9 |
| NPZ-4 score | 0.007 (-0.7 – 0.1)  *n=11* | -0.002 (-0.5 – 0.4)  *n=50* | -0.02 (-0.6 – 0.6)  *n=216* | 0.4 | 0.8 |
| Total PHQ score | 14 (7 – 16)  *n=11* | 12 (7 – 15)  *n=50* | 9 (6 – 14)  *n=216* | 0.2 | 0.09 |
| Abbreviations: ART, anti-retroviral therapy; HCV, hepatitis C virus; anti-HBs, hepatitis B surface antibody; HBsAg, hepatitis B surface antigen; VDRL, Venereal Disease Research Laboratory test; NPZ-4, neuropsychological test z-score in four domains (Color Trails 1, Color Trails 2, Trail Making A, Grooved Pegboard); PHQ, Patient Health Questionnaire. ^†^P-value is for suboptimal recovery vs complete recovery.  ^‡^P-value is for combined suboptimal & intermediate recovery vs complete recovery. | | | | | |

**Supplemental Table 3.**

| Post-ART Variables | Suboptimal Recovery, CD4<350 (n=11) | Suboptimal & Intermediate Recovery, CD4<500 (n=55) | Complete Recovery, CD4≥500 (n=249) | p-value^†^ | p-value^‡^ |
| --- | --- | --- | --- | --- | --- |
| Positive anti-HCV, *n* (%) | 0/2 (0) | 1/15 (7) | 1/49 (2) | 0.8 | 0.4 |
| Positive anti-HBs, *n* (%) | 2/2 (100) | 14/16 (88) | 42/50 (84) | 0.5 | 0.7 |
| Positive HBsAg, *n* (%) | 0/2 (0) | 1/16 (6) | 8/49 (16) | 0.5 | 0.3 |
| Reactive VDRL, *n* (%) | 0/1 (0) | 4/9 (44) | 16/40 (40) | 0.4 | 0.8 |
| NPZ-4 score at week 96 | 0.6 (-0.04 – 0.8)  *n=7* | 0.5 (-0.04 – 0.9)  *n=33* | 0.5 (0.03 – 1)  *n=183* | 0.6 | 0.6 |
| Total PHQ score at week 96 | 5 (1 – 8)  *n=7* | 4 (1 – 8)  *n=33* | 5 (2 – 8)  *n=183* | 0.9 | 0.4 |
| Abbreviations: ART, anti-retroviral therapy; HCV, hepatitis C virus; anti-HBs, hepatitis B surface antibody; HBsAg, hepatitis B surface antigen; VDRL, Venereal Disease Research Laboratory test; NPZ-4, neuropsychological test z-score in four domains (Color Trails 1, Color Trails 2, Trail Making A, Grooved Pegboard); PHQ, Patient Health Questionnaire. ^†^P-value is for suboptimal recovery vs complete recovery.  ^‡^P-value is for combined suboptimal & intermediate recovery vs complete recovery. | | | | | |

**Supplemental Table 4.**

|  | 2NRTI/LPV/r | 2NRTI/EFV | 2NRTI/RAL | 2NRTI/MVC/RAL | 2NRTI/EFV/MVC/RAL | **Total** |
| --- | --- | --- | --- | --- | --- | --- |
| Suboptimal Recovery, CD4<350 | 0 | 8 | 0 | 1 | 2 | 11 |
| Intermediate Recovery, 350≤CD4<500 | 0 | 29 | 0 | 0 | 15 | 44 |
| Complete Recovery, CD4≥500 | 1 | 187 | 1 | 0 | 60 | 249 |
| **Total** | 1 | 224 | 1 | 1 | 77 | 304 |
| Abbreviations: NRTI, nucleoside reverse transcriptase inhibitor; LPV, lopinavir; r, ritonavir; EFV, efavirenz; RAL, raltegravir; MVC, maraviroc. Fisher’s exact test, p=0.1. | | | | | | |

**Supplemental Table 5.**

|  |  | Univariate | | Multivariate | | |
| --- | --- | --- | --- | --- | --- | --- |
| Characteristic | n/N (%) | OR (95% CI) | p-value | OR (95% CI) | p-value | |
| Baseline CD4 count  <350 cells/mm^3^  350-499 cells/mm^3^  ≥500 cells/mm^3^ | 135/304 (44)  92/304 (30)  77/304 (25) | 7.85 (2.69 – 22.94)  2.41 (0.74 – 7.92)  1 (ref) | < 0.001  0.002  0.001 | 6.26 (2.02 – 19.71)  1.30 (0.35 – 4.78)  1 (ref) | <0.001  0.001  0.693 | |
| ART duration  48-119 weeks  120-156 weeks  >156 weeks | 118/304 (39)  67/304 (22)  119/304 (39) | 1.75 (0.91 – 3.37)  0.87 (0.37 – 2.06)  1 (ref) | 0.126  0.096  0.753 | 1.23 (0.55 – 2.78)  0.62 (0.23 – 1.72)  1 (ref) | 0.362  0.611  0.361 | |
| Baseline HIV-RNA  <10^6^ copies/mm^3^  ≥10^6^ copies/mm^3^ | 177/304 (58)  127/304 (42) | 1 (ref)  1.72 (0.96 – 3.09) | 0.071 |  |  | |
| Baseline CD4/CD8 ratio  <1  ≥1 | 214/304 (70)  90/304 (30) | 3.43 (1.49-7.91)  1 (ref) | 0.004 | 5.62 (1.99 – 15.89)  1 (ref) | 0.001 | |
| Baseline platelet count  <300,000 per mm^3^  ≥300,000 per mm^3^ | 248/304 (82)  56/304 (18) | 4.69 (1.41-15.60)  1 (ref) | 0.012 |  |  | |
| On-ART CD4/CD8 ratio  <1  ≥1 | 116/304 (38)  188/304 (62) | 2.73 (1.50-4.96)  1 (ref) | 0.001 |  |  | |
| On-ART CD8 count  <500 cells/mm^3^  ≥500 cells/mm^3^ | 98/304 (32)  206/304 (68) | 3.19 (1.75-5.82)  1 (ref) | < 0.001 | 4.15 (1.94 – 8.88)  1 (ref) | <0.001 | |
| On-ART hemoglobin  <14.8 mg/dL  ≥14.8 mg/dL | 151/283 (53)  132/283 (47) | 1 (ref)  2.04 (1.09-3.84) | 0.026 |  |  | |
| On-ART platelet count  <300,000/mm^3^  ≥300,000/mm^3^ | 210/284 (74)  74/284 (26) | 3.66 (1.39-9.62)  1 (ref) | 0.009 | 2.94 (1.05 – 8.22)  1 (ref) | 0.040 | |
| Baseline I-FABP  <1000 pg/mL  ≥1000 pg/mL | 35/71 (49)  36/71 (51) | 8.75 (1.02-75.37)  1 (ref) | 0.048 |  |  | |
| Baseline CSF neopterin  <1600 pg/mL  ≥1600 pg/mL | 35/79 (44)  44/79 (56) | 1 (ref)  8.74 (1.05-72.78) | 0.045 |  |  | |
| sCD14 at week 96  <1.8 µg/mL  ≥1.8 µg/mL | 26/28 (93)  2/28 (7) | 1 (ref)  12.0 (0.53-273.04) | 0.119 |  |  | |
| IL6 at week 96  <0.2 pg/mL  ≥0.2 pg/mL | 26/74 (35)  48/74 (65) | 6.90 (1.28-37.18)  1 (ref) | 0.025 |  |  | |
| We used multivariate logistic regression models to adjust for duration of ART, baseline HIV-RNA, and baseline CD4 count. 284 participants (93% of 304) were included in the final model. After adjustment, odds of poor CD4 recovery were higher in participants with baseline CD4/CD8 ratio < 1 (odds ratio [OR] 5.62, 95% confidence interval [CI] 1.99-15.89, p=0.001), on-ART CD8 count < 500 cells/mm^3^ (OR 4.15, 95% CI 1.94-8.88, p<0.001), and on-ART platelet count < 300,000 per mm^3^ (OR 2.94, 95% CI 1.05-8.22, p=0.04). | | | | | |  |

**Supplemental Figure 1. Longitudinal HIV-RNA of participants with acute HIV infection.**

**Supplemental Figure 2. Longitudinal CD4+ T cell counts of participants with acute HIV infection according to low, medium, and high baseline CD4 count.** (A) Mean CD4+ T cell counts of all participants (black) and by low (red, baseline CD4 < 350 cells/mm^3^), medium (green, baseline CD4 350-499 cells/mm^3^), and high (blue, baseline CD4 ≥ 500 cells/mm^3^) baseline CD4 count. Dotted black line represents the total number of participants at each study visit week. B-D, CD4+ T cell counts of participants with low (B), medium (C), and high (D) baseline CD4 count. Solid lines represent the mean CD4+ T cell count at each follow-up study visit week. Dashed lines represent the 95% confidence interval of CD4+ T cell counts at each study visit week. Dotted black lines represent the total number of participants in each respective group at each study visit week.

**Supplemental Figure 3. Proportions of low, medium, and high concurrent CD4+ T cell counts of participants with acute HIV infection.** Proportions are displayed at intervals of 24 weeks. Numbers above bars represent the number of participants at the respective study visit week.
